# Supplementary material for: Molecular subtyping based on hippocampal cryptic exon burden reveals proteome-wide changes associated with TDP-43 pathology across the spectrum of LATE and Alzheimer’s Disease
Source: bioRxiv. 2025 Jul 2:2025.05.30.656396. Originally published 2025 Jun 3. Preprint. [Version 2] doi: 10.1101/2025.05.30.656396 (PMC12157639; doi:10.1101/2025.05.30.656396)

bioRxiv preprint doi: https://doi.org/10.1101/2025.05.30.656396; this version posted July 2, 2025. The copyright holder for this preprint (which was not certified by peer review) is the author/funder, who has granted bioRxiv a license to display the preprint in perpetuity. It is made available under aCC-BY 4.0 International license.

Supplemental Figure 1

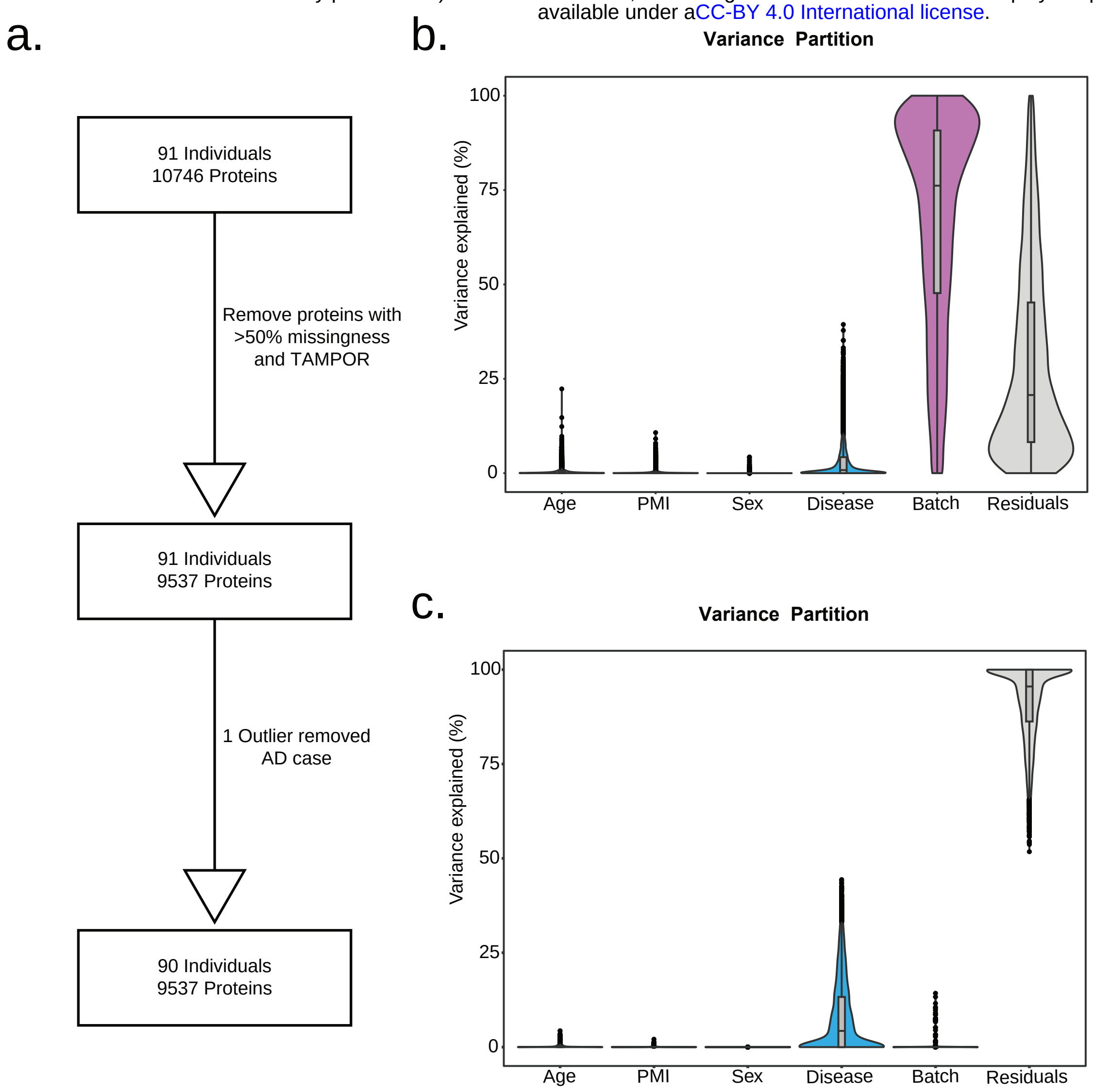

bioRxiv preprint doi: https://doi.org/10.1101/2025.05.30.656396; this version posted July 2, 2025. The copyright holder for this preprint (which was not certified by peer review) is the author/funder, who has granted bioRxiv a license to display the preprint in perpetuity. It is made available under aCC-BY 4.0 International license.

Supplemental Figure 2

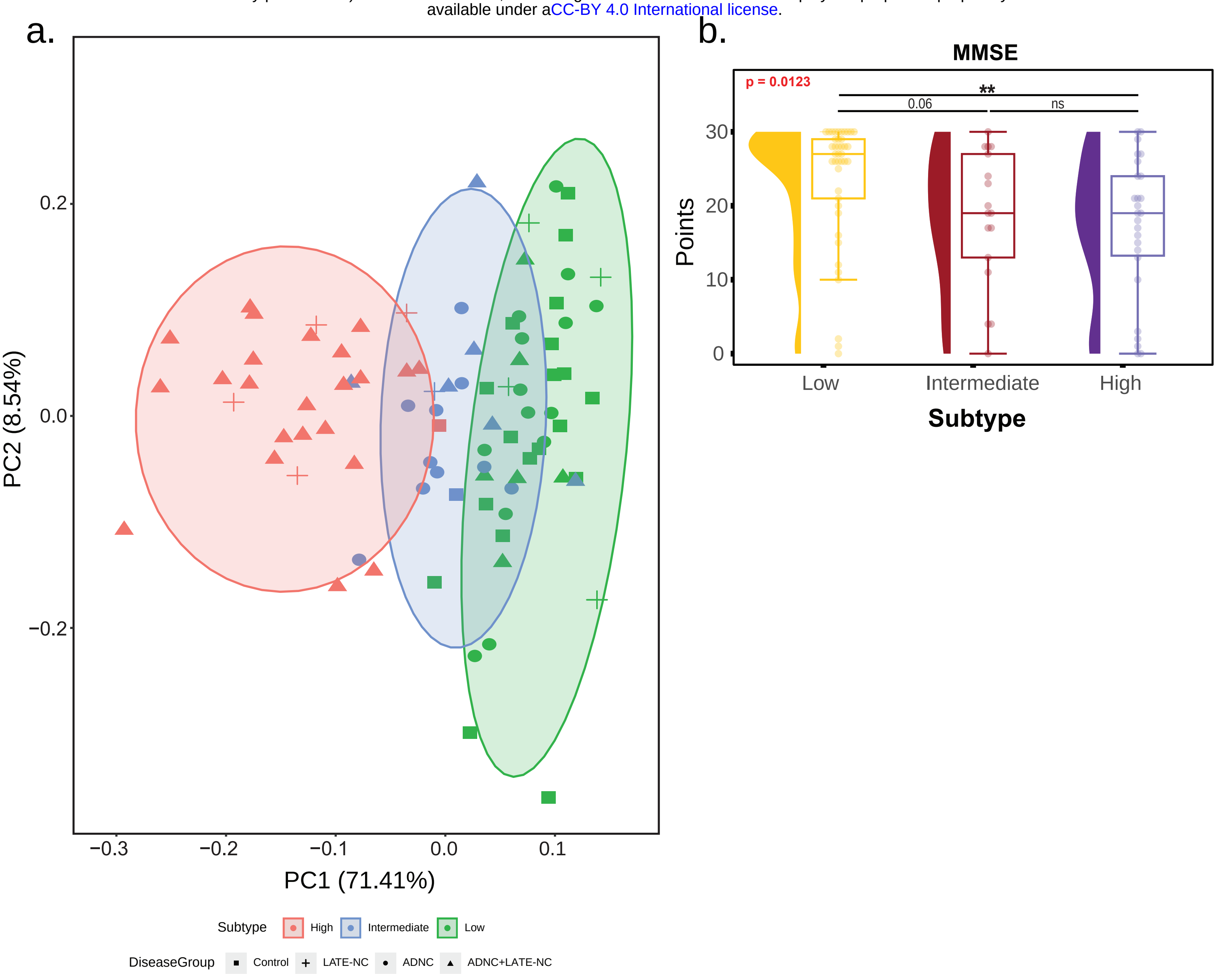

bioRxiv preprint doi: https://doi.org/10.1101/2025.05.30.656396; this version posted July 2, 2025. The copyright holder for this preprint (which was not certified by peer review) is the author/funder, who has granted bioRxiv a license to display the preprint in perpetuity. It is made available under aCC-BY 4.0 International license.

Supplemental Figure 3

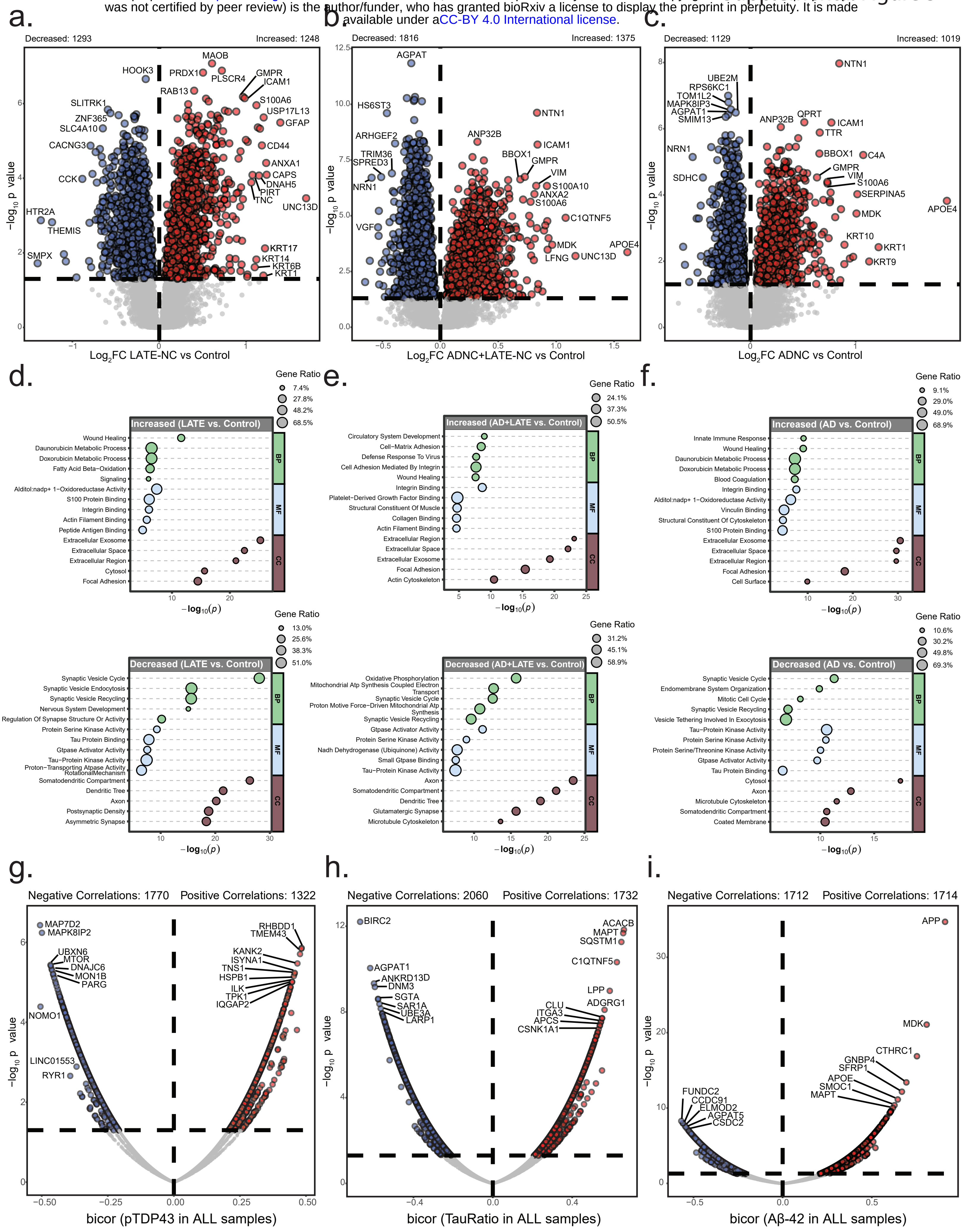

bioRxiv preprint doi: https://doi.org/10.1101/2025.05.30.656396; this version posted July 2, 2025. The copyright holder for this preprint (which was not certified by peer review) is the author/funder, who has granted bioRxiv a license to display the preprint in perpetuity. It is made available under aCC-BY 4.0 International license.

Supplemental Figure 4

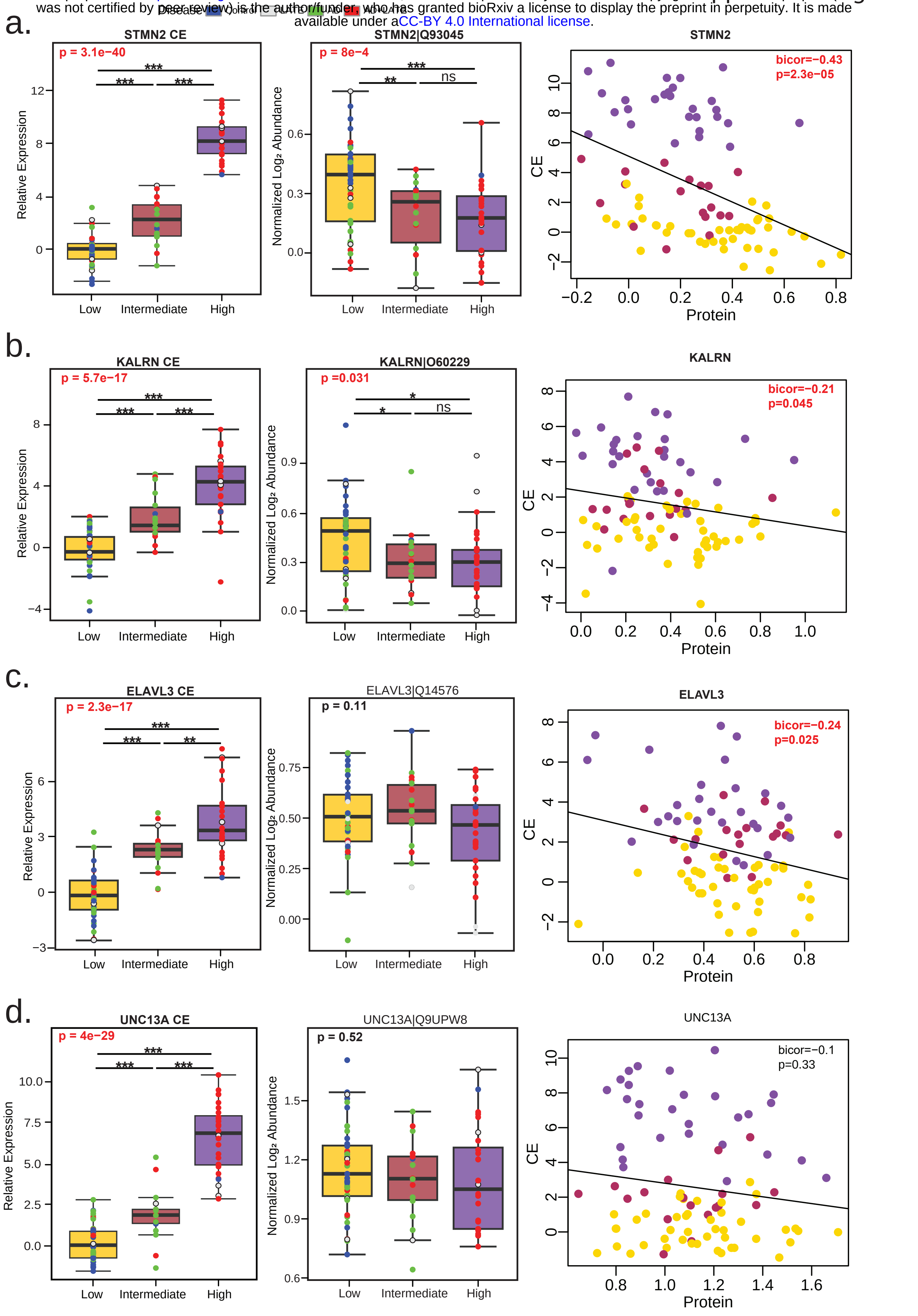

bioRxiv preprint doi: https://doi.org/10.1101/2025.05.30.656396; this version posted July 2, 2025. The copyright holder for this preprint (which was not certified by peer review) is the author/funder, who has granted bioRxiv a license to display the preprint in perpetuity. It is made available under aCC-BY 4.0 International license.

Supplemental Figure 5

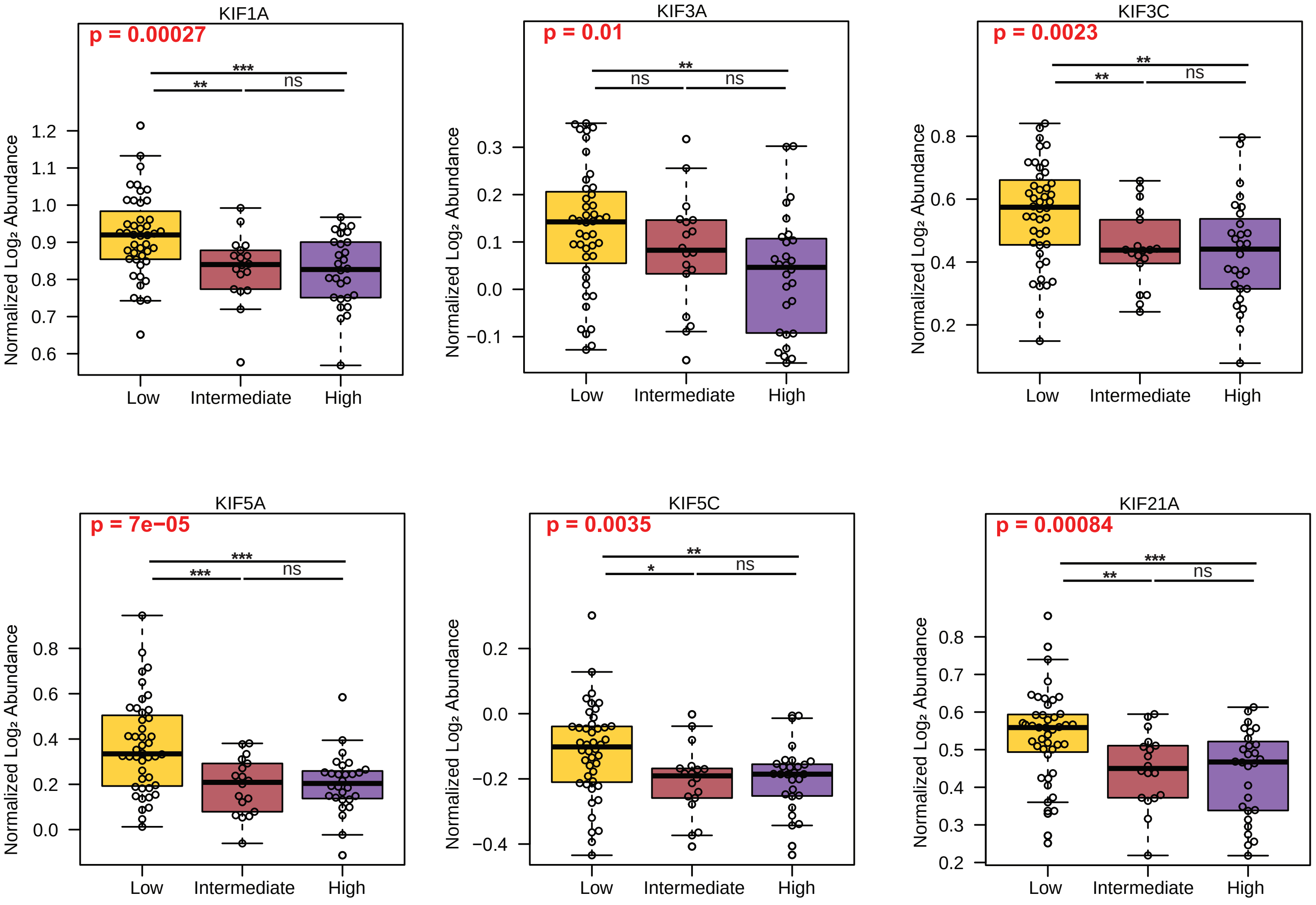

bioRxiv preprint doi: https://doi.org/10.1101/2025.05.30.656396; this version posted July 2, 2025. The copyright holder for this preprint (which was not certified by peer review) is the author/funder, who has granted bioRxiv a license to display the preprint in perpetuity. It is made available under aCC-BY 4.0 International license.

Supplemental Figure 6

a.

GENE	Peptide ID	Charge State	Gradient (min)	PEP	Qvalue	Total Quantity
SYT7	_AINFPGARLQSAA	2	5	2.82E-07	6.7E-07	23381.63
DNM1	_APPGVPRPAPPVR	3	5	2.66E-12	6.41E-12	1496.72
HDGFL2	_GHSGMLASEGREAVLTR	3	5	1.03E-07	2.34E-05	177.87
HDGFL2	_GHSGMLASEGREAVLTR	4	5	6.53E-07	1.06E-06	201.01
RSF1	_VLQAPPPDVGNGEGSR	2	5	3.47E-12	1.34E-07	29.63
MYO18A	_EEDKTLPK	2	30	1.53E-05	5.22E-08	50.74

b.

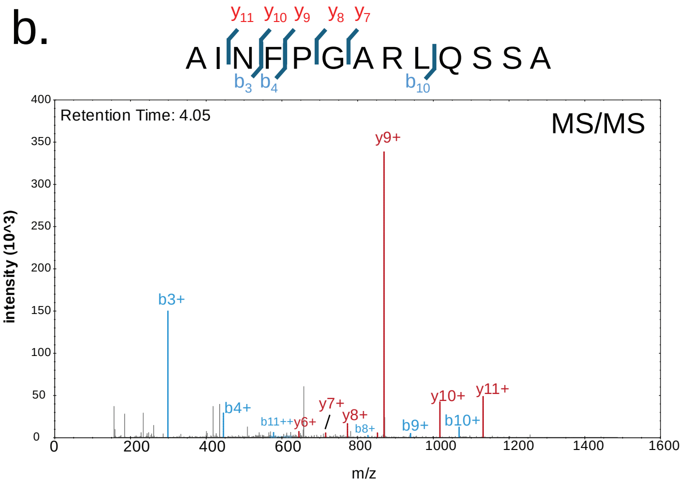

c.

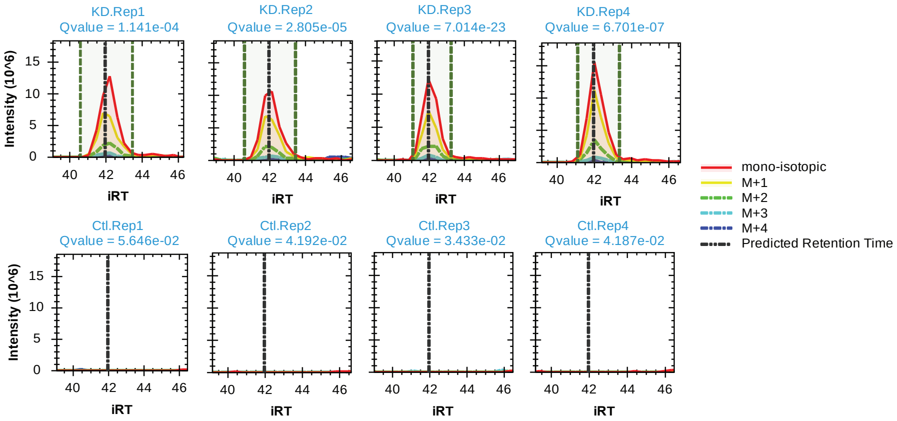

d.

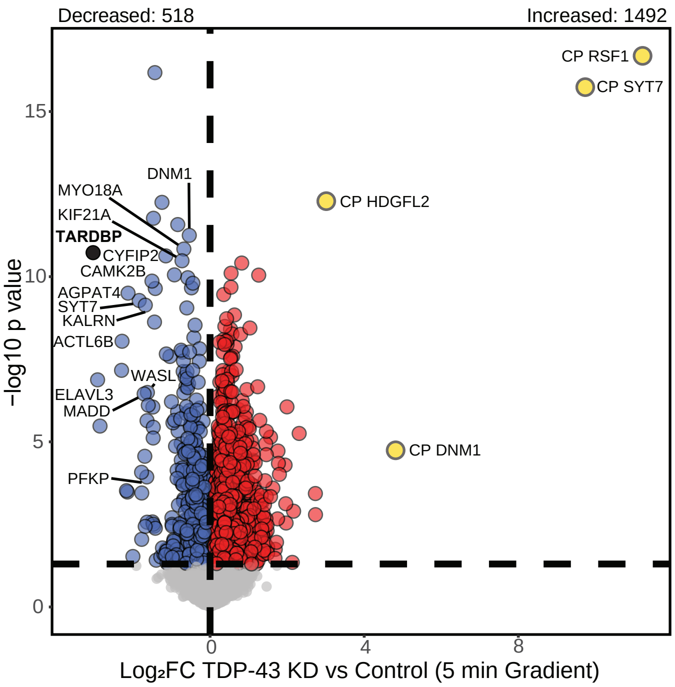

e.

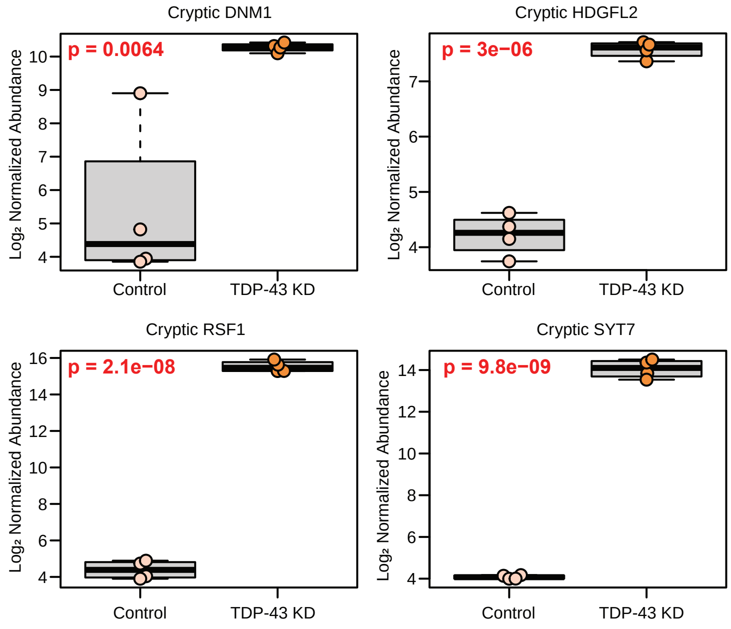

Supplement: 1 — Supplemental Figure 1. Batch correction and normalization of hippocampus proteomic dataset. A. Experimental design outlining quality control steps, including reductions in sample size and protein count. A variance partition analysis identifies proteins influenced by technical artifacts. B. Median centering of TMT-MS data after TAMPOR processing reduces batch effects. C. Post-regression processing removes batch effects and additionally regresses out age, sex, and postmortem interval (PMI). Supplemental Figure 2. Cryptic exon (CE) profiles resolve molecular subtypes associated with cognition. A. Principal Component Analysis (PCA) shows clear separation between high and low CE burden groups, with intermediates showing mixed profiles. Ellipses represent author added emphasis. B. MMSE scores are compared across subtypes using rainfall plots, including boxplots and score distributions. One-way ANOVA p-values are bolded in red; t-test significance is indicated by asterisks (*p < 0.05, **p < 0.01, ***p < 0.001). Supplemental Figure 3. Disease-specific proteomic signatures across clinicopathological subgroups. A–C. Volcano plots of differentially abundant proteins between LATE-NC, ADNC+LATE-NC, ADNC, and control hippocampus samples. Red = increased; blue = decreased; grey = non-significant. D–F. Gene Ontology (GO) enrichment for upregulated (top) and downregulated (bottom) proteins. Circle size represents gene ratio. GO terms categorized by Biological Process (BP), Molecular Function (MF), and Cellular Component (CC). G–I. BiCor correlations between individual proteins and key ADNC/LATE-NC biomarkers (pTDP-43, pTau/Tau, Aβ42). Top correlated proteins are labeled. Supplemental Figure 4. Inverse relationship between cryptic exon (CE) abundance and protein levels. Boxplots and scatterplots show cryptic exon burden, total protein abundance, and their relationship for STMN2, KALRN, ELAVL3, and UNC13A. Points are colored by diagnosis. ANOVA p-values are bold and red where CE burde [file NIHPP2025.05.30.656396V2-supplement-1.pdf]
