# Supplementary material for: Molecular Subtyping Based on Hippocampal Cryptic Exon Burden Reveals Proteome-wide Changes Associated with TDP-43 Pathology across the Spectrum of LATE and Alzheimer’s Disease
Source: bioRxiv. 2025 Jun 3:2025.05.30.656396. Preprint. [Version 1] doi: 10.1101/2025.05.30.656396 (PMC12157639; doi:10.1101/2025.05.30.656396)

bioRxiv preprint doi: https://doi.org/10.1101/2025.05.30.656396; this version posted June 3, 2025. The copyright holder for this preprint (which was not certified by peer review) is the author/funder, who has granted bioRxiv a license to display the preprint in perpetuity. It is made available under aCC-BY 4.0 International license.

Supplemental Figure 1

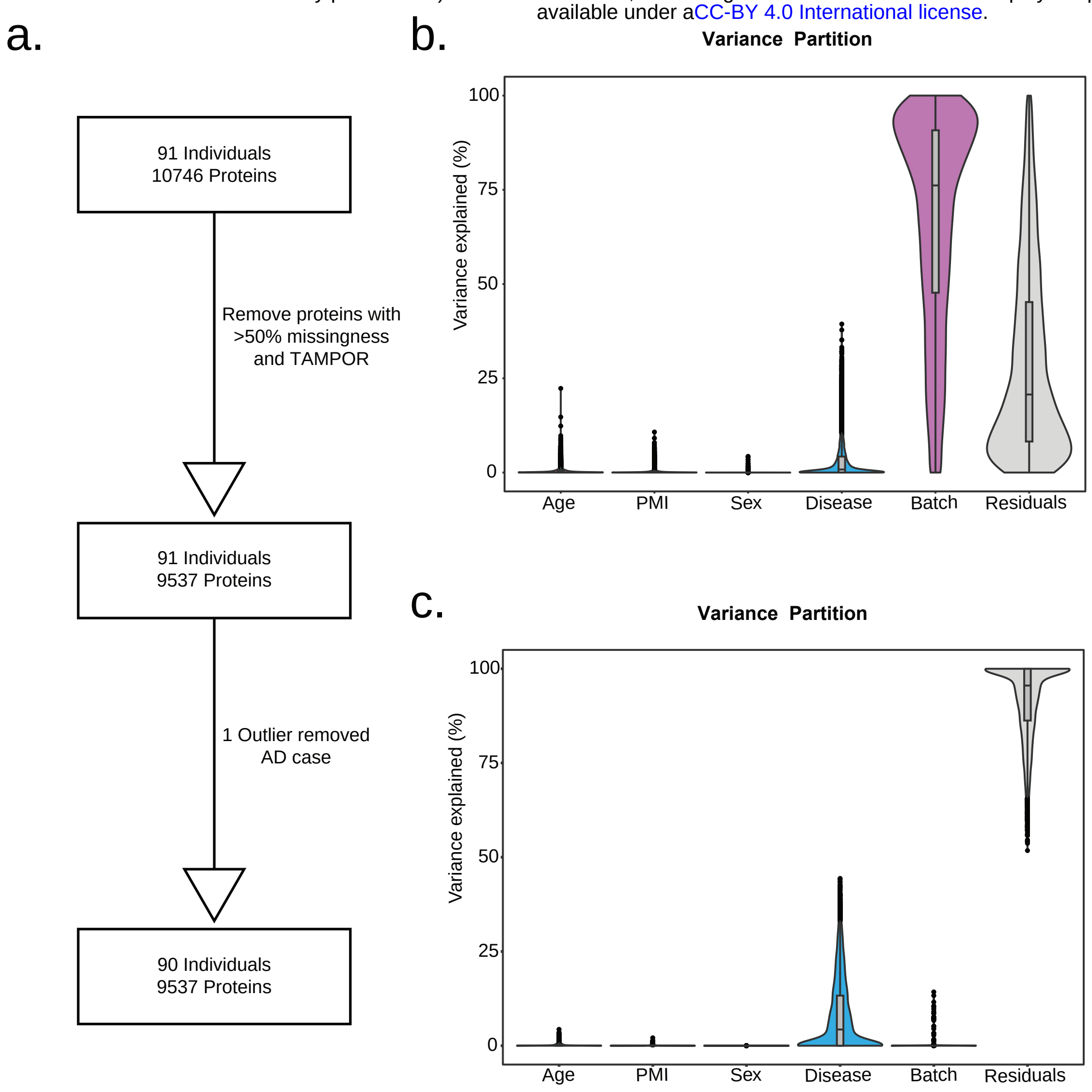

bioRxiv preprint doi: https://doi.org/10.1101/2025.05.30.656396; this version posted June 3, 2025. The copyright holder for this preprint (which was not certified by peer review) is the author/funder, who has granted bioRxiv a license to display the preprint in perpetuity. It is made available under aCC-BY 4.0 International license.

Supplemental Figure 2

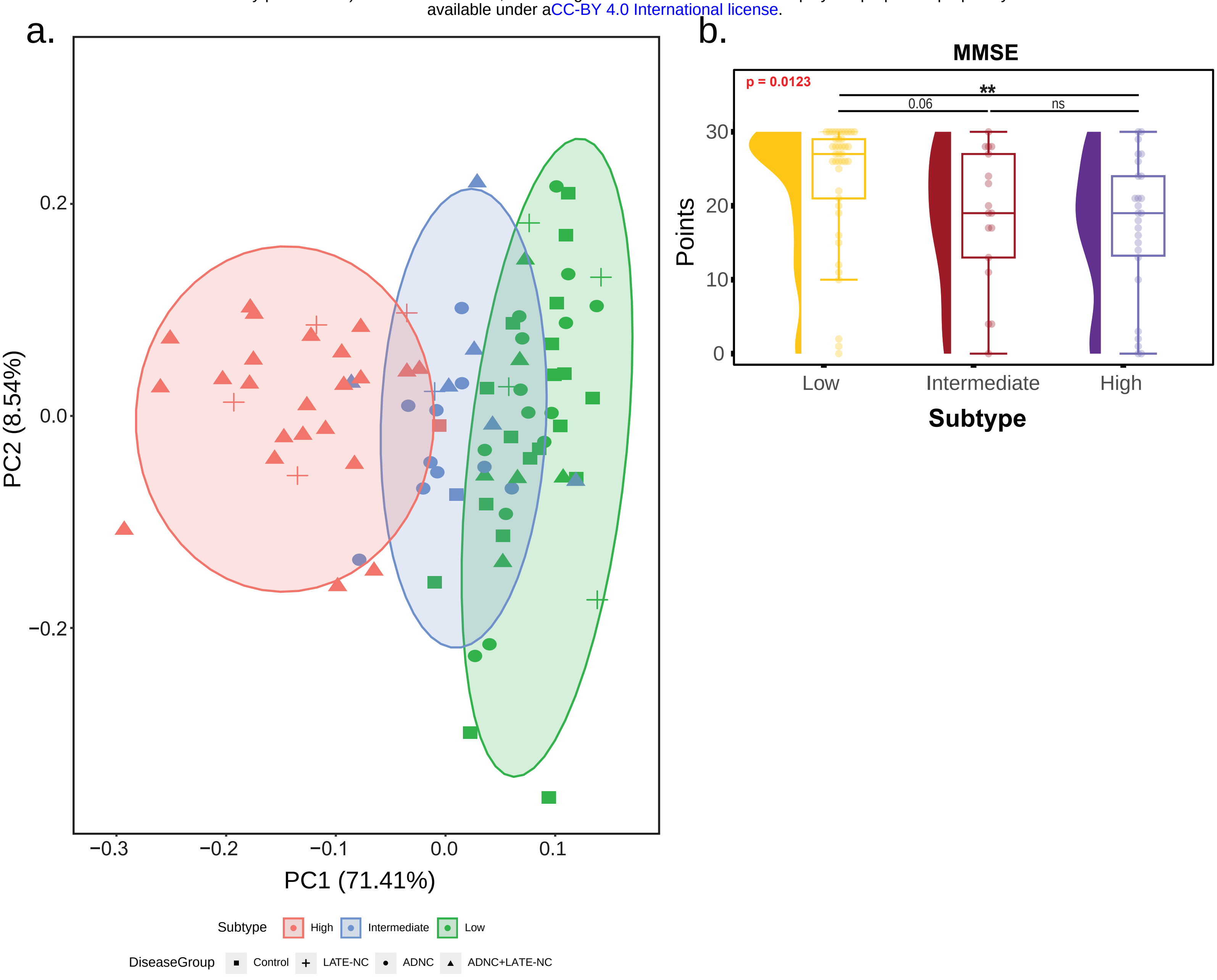

bioRxiv preprint doi: https://doi.org/10.1101/2025.05.30.656396; this version posted June 3, 2025. The copyright holder for this preprint (which was not certified by peer review) is the author/funder, who has granted bioRxiv a license to display the preprint in perpetuity. It is made available under aCC-BY 4.0 International license.

Supplemental Figure 3

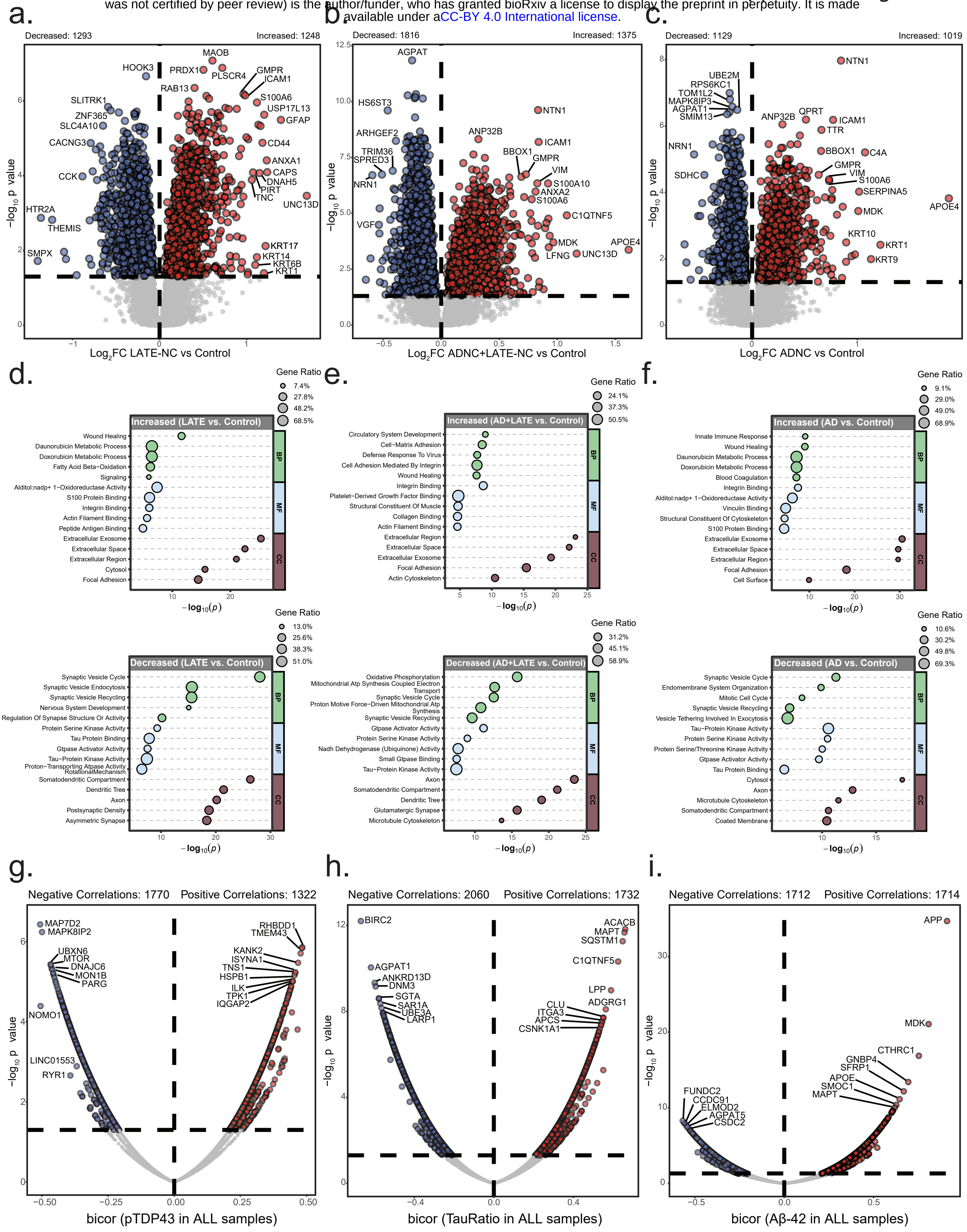

bioRxiv preprint doi: https://doi.org/10.1101/2025.05.30.656396; this version posted June 3, 2025. The copyright holder for this preprint (which was not certified by peer review) is the author/funder, who has granted bioRxiv a license to display the preprint in perpetuity. It is made available under aCC-BY 4.0 International license.

Supplemental Figure 4

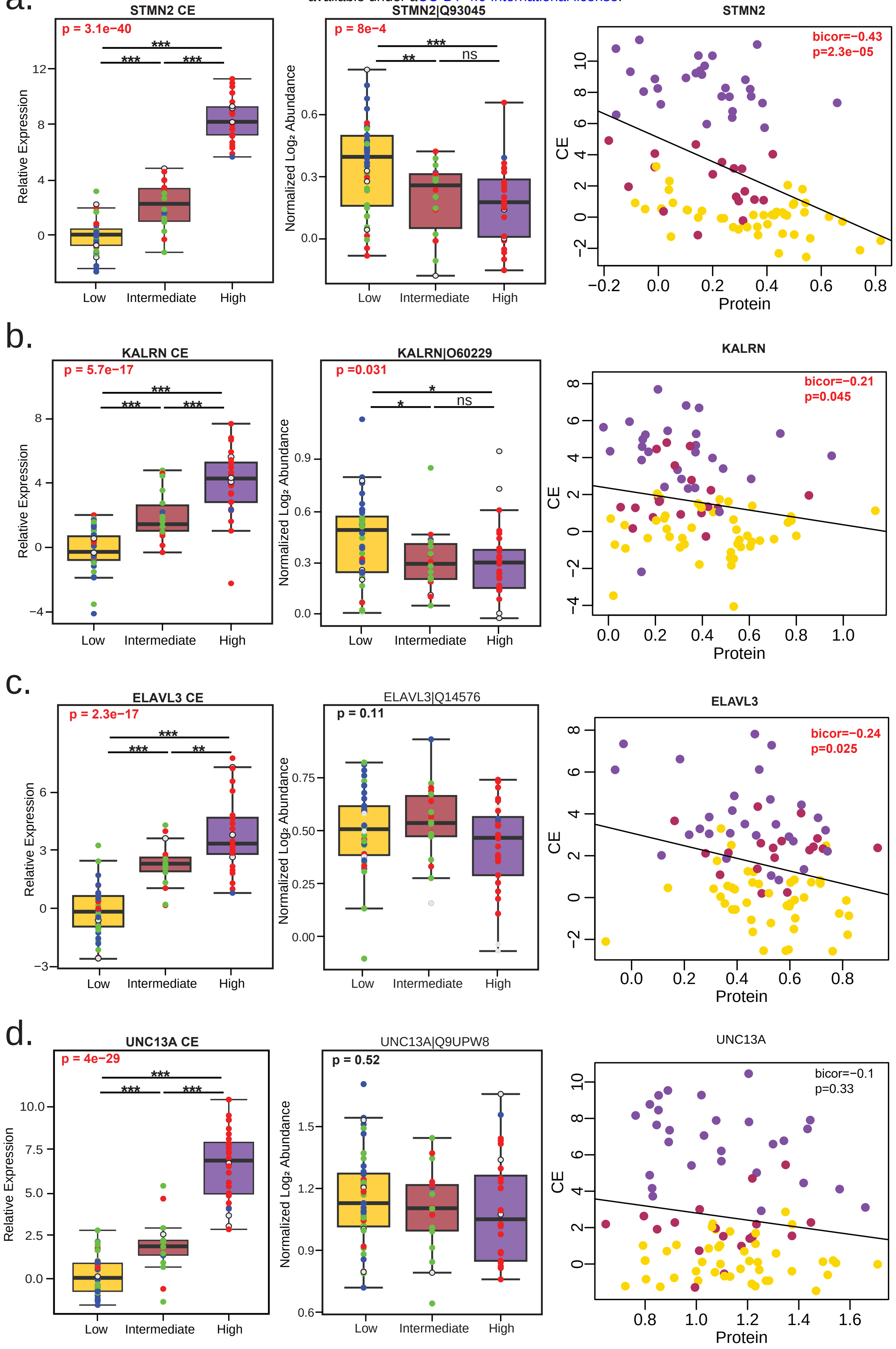

bioRxiv preprint doi: https://doi.org/10.1101/2025.05.30.656396; this version posted June 3, 2025. The copyright holder for this preprint (which was not certified by peer review) is the author/funder, who has granted bioRxiv a license to display the preprint in perpetuity. It is made available under aCC-BY 4.0 International license.

# Supplemental Figure 5

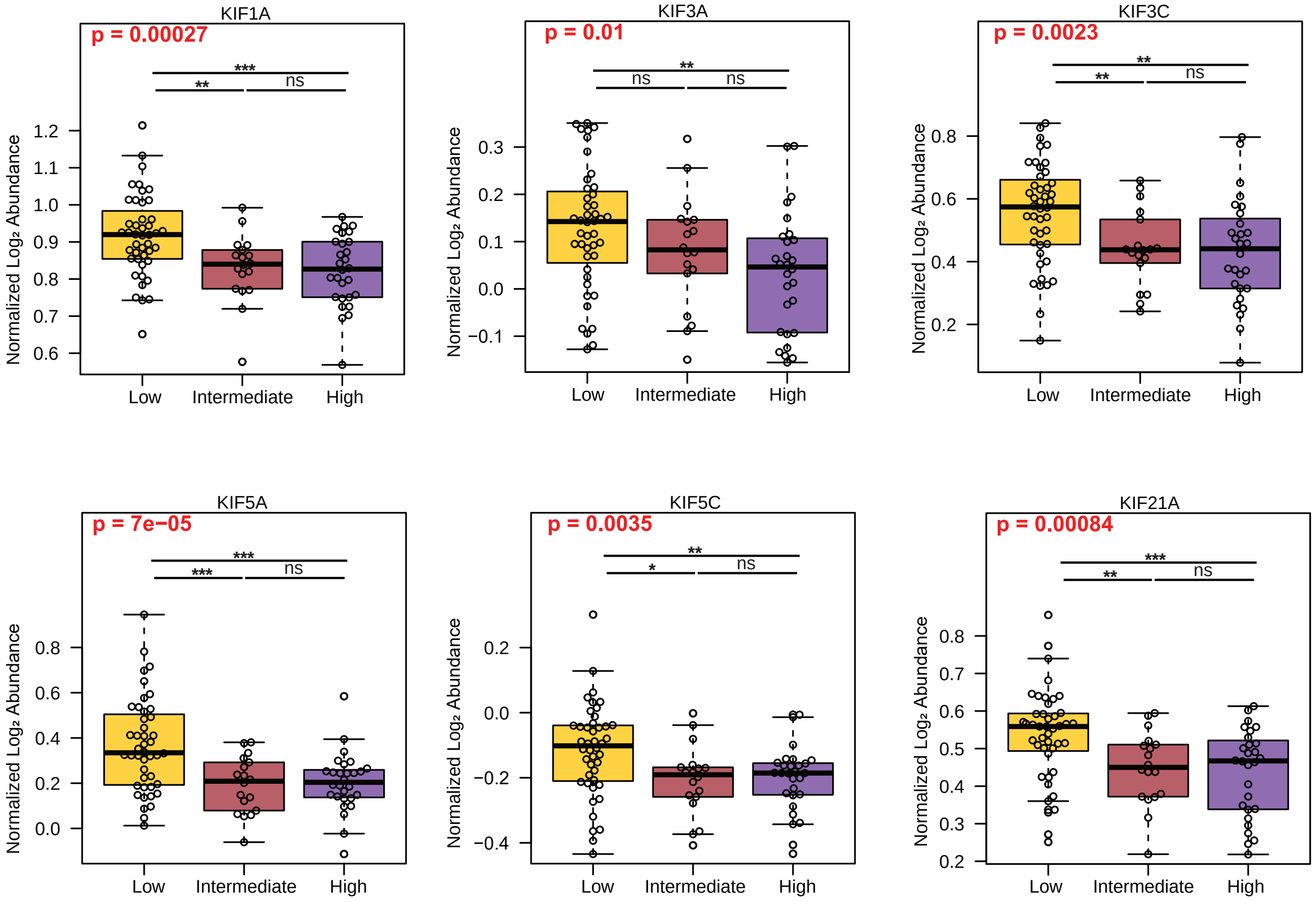

bioRxiv preprint doi: https://doi.org/10.1101/2025.05.30.656396; this version posted June 3, 2025. The copyright holder for this preprint (which was not certified by peer review) is the author/funder, who has granted bioRxiv a license to display the preprint in perpetuity. It is made available under aCC-BY 4.0 International license.

Supplemental Figure 6

a.

| GENE | Peptide ID | Charge State | Gradient (min) | PEP | Qvalue | Total Quantity |
|---|---|---|---|---|---|---|
| SYT7 | _AINFPGARLQSAA | 2 | 5 | 2.82E-07 | 6.7E-07 | 23381.63 |
| DNM1 | _APPGVPRPAPPVR | 3 | 5 | 2.66E-12 | 6.41E-12 | 1496.72 |
| HDGFL2 | _GHSGMLASEGREAVLTR | 3 | 5 | 1.03E-07 | 2.34E-05 | 177.87 |
| HDGFL2 | _GHSGMLASEGREAVLTR | 4 | 5 | 6.53E-07 | 1.06E-06 | 201.01 |
| RSF1 | _VLQAPPPDVGNGEGSR | 2 | 5 | 3.47E-12 | 1.34E-07 | 29.63 |
| MYO18A | _EEDKTLPK | 2 | 30 | 1.53E-05 | 5.22E-08 | 50.74 |

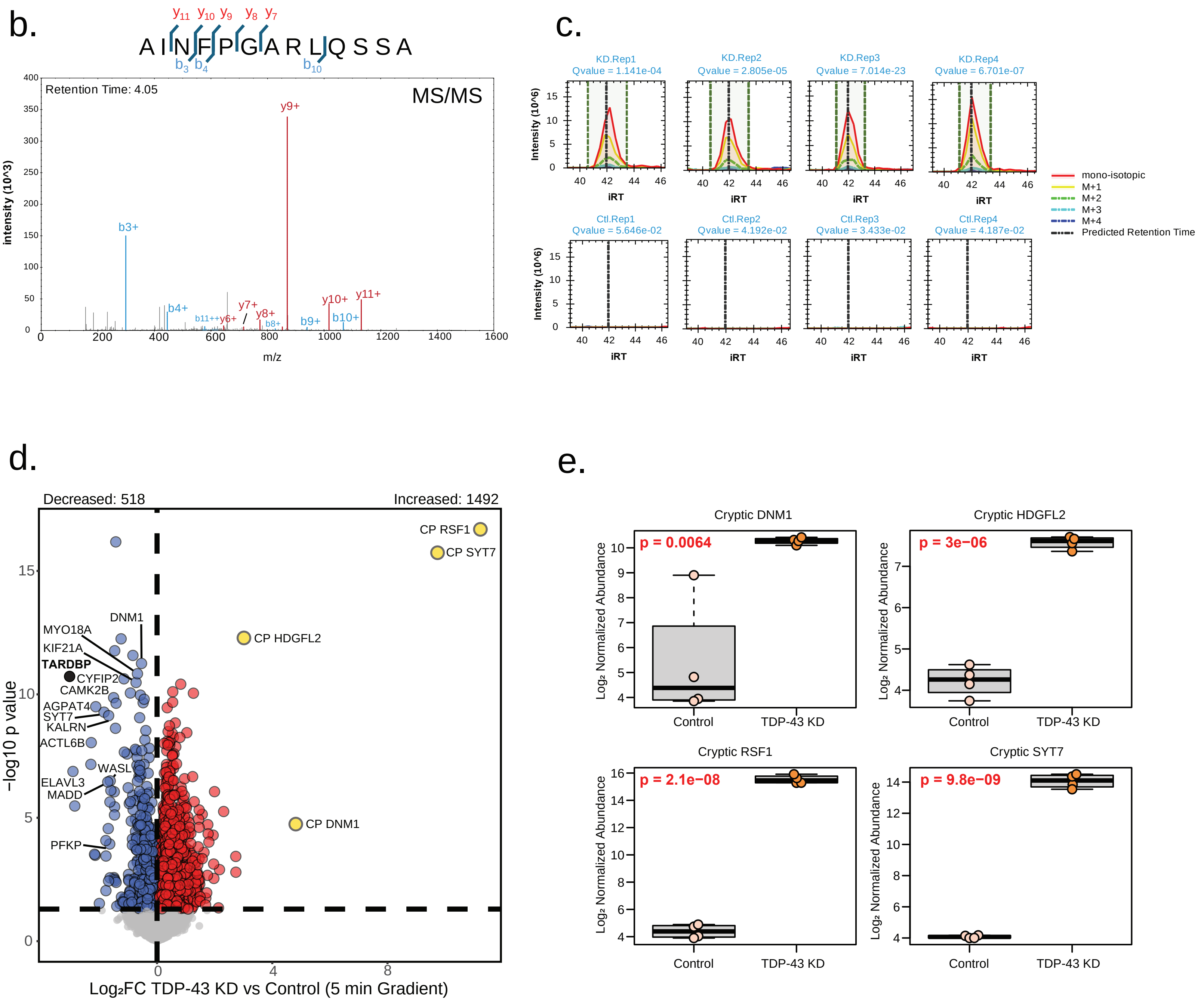

Supplement: Supplementary file 1 [file NIHPP2025.05.30.656396V1-supplement-1.pdf]
